# Comparing water-based and land-based exercise on 6-minute run test performance in overweight adults: A randomized controlled trial in Germany

**DOI:** 10.1016/j.pmedr.2025.103279

**Published:** 2025-10-11

**Authors:** Vanessa Maria Eichel, Katja Oomen-Welke, Maria Störk, Roman Huber, Maximilian Andreas Storz, Johannes Naumann

**Affiliations:** a Center for Complementary Medicine, Medical Center - University of Freiburg, Faculty of Medicine, University of Freiburg, Hugstetterstr.55, Freiburg im Breisgau 79106, Germany; b European Institute for Physical Therapy and Balneology (EIPB), Sonnenbergstr.35, Freiburg 79117, Germany

**Keywords:** Aqua-cycling, Balneotherapy, Cardiovascular risk factors, Run-test, Aquatic exercise, Water-based exercise, Overweight

## Abstract

**Objective:**

This study compared the effects of water-based exercise (WBE) and land-based exercise (LBE) on 6-min run test (6MRT) performance in overweight adults (BMI ≥ 25 kg/m^2^) and examined changes in cardiovascular risk factors.

**Methods:**

Participants were randomly assigned (1:1:1) to WBE (aqua-cycling), LBE (fitness) or a control group (CLG, no training). Sessions lasted 45 min, twice weekly, over 10 weeks (2018 professional facilities, Freiburg, Germany). The primary endpoint was change in 6MRT from baseline to post-intervention; secondary endpoints included hip-belly ratio (HBR), body mass index (BMI), and cardiovascular risk factors. Baseline assessments were double-blinded; 6MRT was analyzed single-blind.

**Results:**

Eightly-seven participants (56 female, age 52.2 ± 12.3 years, BMI 33.9 ± 4.95) were enrolled.Both exercise groups improved 6MRT, HBR, and BMI, but only LBE showed a significant advantage over CLG in 6MRT (*p* < 0.05). No significant differences were observed between WBE and LBE. Cardiovascular risk factors (blood pressure, lipids, HbA1c) did not differ significantly across groups.

**Conclusions:**

WBE and LBE improved fitness and body composition, but only LBE achieved a significant gain versus control. No exercise modality differentially influenced cardiovascular risk factors. Choice between WBE and LBE could be guided by preference, access, and functional needs.

## Highlights

• Water- and land-based exercise were compared in overweight adults.
• Randomized controlled trial with 87 participants in three groups.
• Both exercise types improved fitness, body mass index, and waist-hip ratio.
• Land-based exercise improved six-minute run compared with control.
• Participants preferred water-based exercise despite smaller fitness gains.

## 1 Introduction

In 2022, 2.5 billion adults were overweight—890 million (16 %) with obesity—a doubling of obesity since 1990. Global Burden of Disease estimates 2.11 billion adults had overweight or obesity in 2021, rising to 3.8 billion by 2050 (Ng et al., 2025; WHO, 2025).

Cardiovascular fitness programs can reduce overweight as well as its risks (Bray et al., 2016; Andrade et al., 2020). The 2025 ACSM survey shows that diverse modalities—resistance training, aerobic exercise, and HIIT—are top fitness trends in overweight populations, and structured exercise safely improves body composition and cardiometabolic health (Batrakoulis et al., 2021; Al-Mhanna et al., 2023; Batrakoulis et al., 2022; Al-Mhanna et al., 2025; Newsome et al., 2024).

Nevertheless, problems with lifestyle change include acceptance and long-term success (Teixeira et al., 2015; MacLean et al., 2015). Compared to land, water offers an increased resistance, buoyancy, and temperature effects. Water's omnidirectional resistance and buoyancy in WBE balance agonist–antagonist activation, correcting compensatory patterns and reducing imbalances (Cuesta-Vargas et al., 2020). Dynamic balance training is effective in water, especially for the elderly (Lord et al., 1993; Bento, and F. A., Cebolla, E. C., Wolf, R., and Rodacki, A. L., 2015). Warm water immersion increases muscle temperature and blood flow while reducing sympathetic activity, leading to decreased muscle stiffness and tension in overweight adults (Hoekstra et al., 2018; Stephens et al., 2018).

It takes more effort for people with excessive weight to exercise and overweight people suffer more often from joint and spine diseases (Allen and Golightly, 2015), which limit physical activity. In water, buoyancy helps to overcome these hurdles (Gobbi et al., 2020) and joints are protected. Even individuals with pronounced pain, as in back pain or fibromyalgia, are able to perform WBE without significant problems (Lima et al., 2013) and a high attendance rate (Abadi et al., 2019). WBE can improve both, cardiovascular and neuromuscular parameters (Lee et al., 2017). Fitness tests showed a significant advantage of WBE compared to land-based exercise (LBE) in overweight elementary students (Lee and Oh, 2014). WBE can reduce weight (Irandoust and Taheri, 2015), abdominal circumference, and fat mass, furthermore, it can improve muscle strength and endurance in both upper and lower limbs (Sajeras, 2019). There is evidence that WBE improves insulin resistance in women (Jones et al., 2009). WBE was significantly more enjoyable than LBE (McNamara et al., 2013) and improved the quality of life significantly (Garrido et al., 2016). On the other hand, there are also studies that show no significant difference between WBE and LBE (Lim et al., 2010; Lopera et al., 2016; Greene et al., 2009).

However, scientific data on efficacy of WBE in overweight people is insufficient. Most trials have a very small sample size, are limited to special groups such as students (Lee and Oh, 2014), or show contradictory results (Meredith-Jones et al., 2011). The purpose of this study was to investigate whether WBE or LBE lead to positive health effects in overweight individuals in comparison to a control group.

## 2 Materials and methods

### 2.1 Study design and participants

The three-arm monocentric intervention study included two interventions (WBE and LBE) and a CLG (go shopping in the city without specific training). The recruitment took place from July 2018 to September 2018 via flyers and press releases. After informed consent, we assigned participants randomly 1:1:1 to the three groups of WBE, LBE, and the CLG. Baseline values and evaluation were double-blinded. A blinded researcher gathered the primary outcome criterion.

The study included individuals aged 18 years and older with a BMI ≥ 25 kg/m^2^ who had no severe medical conditions (e.g. joint diseases, broncho-pulmonary disease, or heart disease) that would prevent participation in exercise training and who had not participated in any structured fitness training in the previous twelve weeks by based on self-report. In the CLG we discouraged additional systematic exercise more frequently than once per week. After the training period all participants were allowed to perform any exercise at any intensity.

Participants with severe physical limitations or medical conditions contraindicating exercise participation; current pregnancy or breastfeeding; participation in other structured weight-loss or fitness programs; inability to provide informed consent or complete study procedures were excluded.

Participants rated intervention preference and motivational reasons—fitness, weight loss, and study participation—on 1 (“unimportant”) to 5 (“very important”) scales at study entry.

### 2.2 Randomization and blinding

A blinded researcher used RandList to create a block-randomized list (120 participants, blocks of 15) and sealed assignments in numbered envelopes. After baseline measurements at T3, participants were allocated; although post-allocation blinding wasn't feasible, both baseline data collection and outcome assessments were conducted by investigators blinded to treatment and participant identities. Throughout the study, personal data were pseudonymized using unique participant codes. Data were stored exclusively on password-protected institutional servers in accordance with the General Data Protection Regulation (GDPR), and only authorized personnel had access.

### 2.3 Ethical approval

The study was conducted in accordance with Good Clinical Practice Guidelines (CPMP/ICH/135/95; Topic E6 (R1); and GCP-V), the Declaration of Helsinki and local laws. The protocol was reviewed and approved by the local Ethics Committee (EK-Freiburg 25/18). The study was prospectively registered in the European Clinical Trials Database (**DRKS00014004**). All participants gave written informed consent. Authors had access to the study data and reviewed and approved the final manuscript.

### 2.4 Intervention and CLG protocol

Interventions started one week after randomization. They took place twice weekly for 10 weeks with one week rest due to holidays, resulting in a total of 20 sessions. Each session lasted 45 min. The exercise volume in this study (90 min per week) was set below the ACSM-recommended 150 min, as it had to be feasible for our target group of previously inactive overweight individuals and the goal was not to implement a full preventive training program but to compare the relative effects of two exercise modalities (WBE and LBE) under standardized conditions. Qualified and experienced exercise instructors from professional providers conducted the indoor training in groups of max. 16 participants in a thermal spa in Bad Krozingen (WBE and LBE), in the fitness studio in Emmendingen (LBE) and a public swimming pool in Kenzingen (WBE), respectively. The WBE group received an aqua-cycling program using aqua-bikes, model “Aquarider®” (AquaKinetics®, Teningen, Germany) in warm water (28 °C in Kenzingen and 34 °C in Bad Krozingen). LBE participants engaged in a mixed land-based routine (e.g., step, machine, and floor exercises targeting quadriceps, glutes, and core in an upright, weight-bearing posture). Both interventions followed the same protocol: a 3 min welcome, 5 min briefing, warm-up, 12 min aerobic phase, 18 min intensive coordinative endurance-strength training, 5 min relaxation/stretching, and 2 min farewell. Intensity was self-rated via the Borg scale (mean and peak) in the first and last sessions, and instructors recorded attendance.

**Fig. 1:**
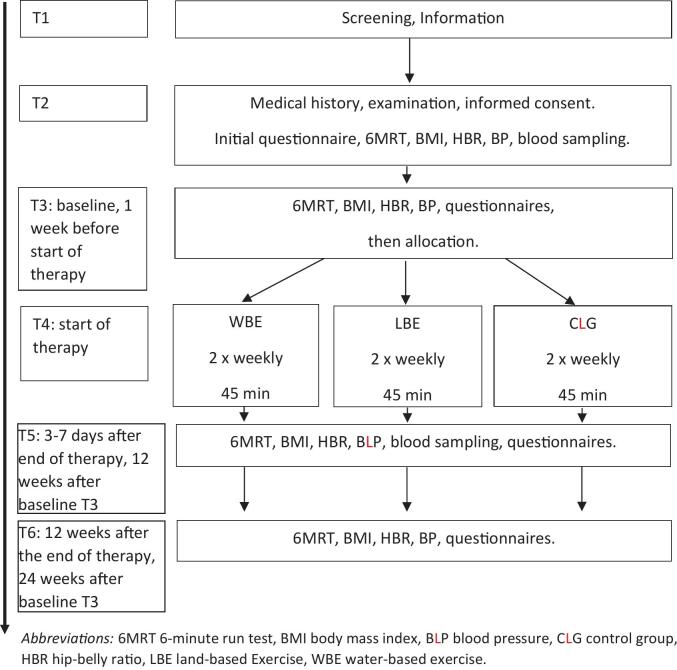
**Time schedule for water-, land-based exercise, and control group in Germany, 2018.***Abbreviations:* 6MRT 6-min run test, BMI body mass index, BLP blood pressure, CLG control group, HBR hip-belly ratio, LBE land-based Exercise, WBE water-based exercise.

The CLG received restaurant vouchers and the request to meander around the city for 45 min twice a week. For the individual participants, the interventions lasted 26 weeks, the whole study (first patient in, last patient out) lasted 43 weeks. The time schedule is displayed in Fig. 1.

### 2.5 Outcome measures

The primary outcome parameter was fitness, measured in terms of how far an individual could move within 6 min (6MRT) (Matabuena et al., 2018a; Matabuena et al., 2018b; Bergmann et al., 2014).We chose the 6MRT—a validated, submaximal field test in overweight adults—to provide a standardized, safe measure of cardiopulmonary fitness across all groups. However, aquatic-specific endurance tests (e.g., pool-based shuttle run or underwater ergometry) might more directly capture WBE adaptations. In order to exclude weather influences, the 6MRT took place in a hall. A marked indoor walking course of known length (e.g. 20–30 m) was used. Participants were instructed to walk as far as possible for six minutes. Timing was recorded with a stopwatch, and usual encouragement was given at regular intervals. Pre- and pos*t*-test perceived exertion was noted using a simple rating scale. We compared between groups the difference of the results measured before (T3) and directly after (T5) the end of the training period. Because the results of the 6MRT gain reliability through prior familiarization (Wu et al., 2003), all individuals had completed the test once before at T2. Secondary outcome parameters were the 6MRT 12 weeks after the end of the training (T6), body mass index (BMI), hip-waist ratio (HBR), blood pressure (BLP), triglycerides (TRG), high density lipoproteins (HDL), low density lipoproteins (LDL), total cholesterol, hemoglobin A1c (HbA1c), quality of life (12-Item Short-Form Health-Survey; SF-12), weight-related quality of life (Impact of Weight on Quality of Life-lite; IWQoL-Lite), International Physical Activity Questionnaire (IPAQ-SF), number of adverse events (AE).

Body mass and stature were measured with calibrated scales and stadiometers, respectively. Seated blood pressure and heart rate were recorded after a period of rest using an automated cuff system. Grip Strength was assessed via a handheld dynamometer with participants performing maximal squeezes. The best of three trials was used.

All secondary outcome parameters were assessed at T3, T5, and T6. We also interviewed individuals about their preference of training type (WBE or LBE), motivation, problems and acceptance.

### 2.6 Sample size calculation

We expected a clinically relevant difference of 15 % of the mean and a SD of 20 % of the mean in the 6MRT test between the WBE and the CLG, as this is seen as a clinically important difference and a typical SD in the 6-min-run-test (Bohannon and Crouch, 2017). With a significance level of *p* < 0.05 and a power of 0.8 in a 2-sided t-test for independent samples this results in *n* = 30 per group (*n* = 90 total). The difference between the two therapy groups was likely to be smaller; here, we took an exploratory approach, for which secondary criteria such as preference, acceptability, and AE or dropout rates were also relevant.

### 2.7 Statistical analyses

After testing the normality of data distribution, we performed ANCOVA after therapy (T5) and group as variable and baseline as cofactor. We report *p*-values with the significance level set at *p* < 0.05. We calculated the effect size using Cohen's d and independent *t*-test between 2 of the 3 groups, regarding a d > 0.5 as effective. Secondary outcomes were predefined as exploratory. No formal adjustment for multiple comparisons was applied; all *p*-values should therefore be regarded as descriptive, and findings should be interpreted cautiously. We analyzed the intention-to-treat (ITT) population, defined as all allocated patients, and applied the last-observation-carried-forward (LOCF) approach to impute missing data. We defined the per-protocol (PP) population as all patients who had a complete dataset for the regarding outcome and had participated in at least 75 % of the treatments, meaning at least 16 of 20 treatments for T5. Statistical analyses were performed using SPSS® (IBM, Version 22).

## 3 Results

### 3.1 Study population

**Fig. 2:**
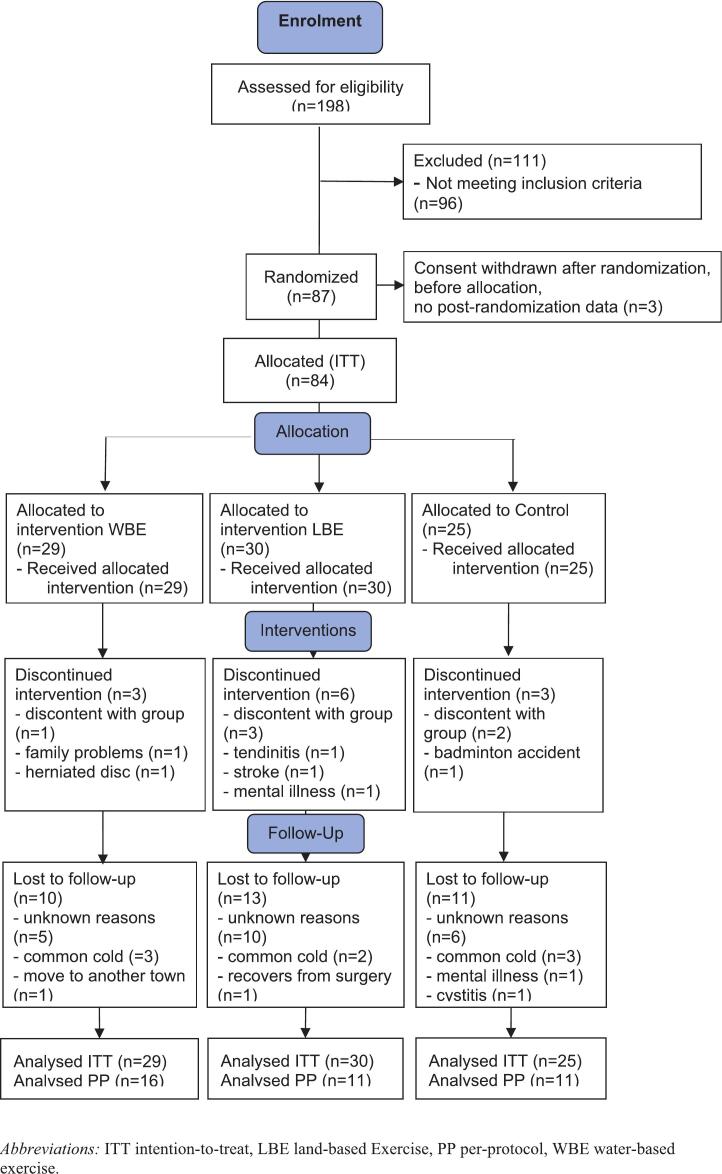
**Flow Diagram of the study course in Germany, 2018.***Abbreviations:* ITT intention-to-treat, LBE land-based Exercise, PP per-protocol, WBE water-based exercise.

Fig. 2 displays the study course, Table 1 shows the demographic characteristics.

**Table 1:** Demographic characteristics of participants of water-, land-based exercise, and control group in Germany, 2018.

	WBE	LBE	CLG	Total
Participants (n)	29	30	25	84
Sex (f/m)	20 / 09	17 / 13	19 / 06	56 / 28
Age (years)	50,7 ± 11,8	52,1 ± 10,5	54,0 ± 14,9	52,2 ± 12,3
Family status (si/mr/ps/di/wd/ot)	6/16/4/3/0/0	5/18/3/1/2/1	5/12/2/5/1/0	16/46/9/9/3/1
Employed (ft/pt./ue/rt)	8/ 13/ 3/ 5	11/ 10 / 4/ 5	6/ 9/ 4/ 6	24/ 32/ 12/ 16
Current smoker (n)	3	3	1	7

Absolute numbers (n) or mean +/− standard deviation.

*Abbreviations*: WBE water-based exercise, LBE land-based exercise, CLG control group, si single, mr married, ps partnership, di divorced, wd widowed, ot others, ft. fulltime (38–40 h/week), pt. parttime (< 38 h/week), ue unemployed, rt. retired.

After screening of 198 prospective participants, we randomized a total of 87 individuals into the three groups using blocks of 15 participants: 29 to WBE, 30 to LBE, and 28 to the CLG. After randomization, but before allocation, 3 CLG participants cancelled their participation. The 84 participants (56 female) had a mean age of 52.2 ± 12.3 years and a BMI of 33.9 ± 4.95. Most were married (46/84) and part-time (32/84) or full-time (24/84) employed.

During interventions, 12 participants dropped out due to illness (5 in total: 1 WBE, 3 LBW, 1 CLG), family reasons (1 WBE) or not satisfied with allocation (6 in total: 1 WBE, 3 LBE, 2 CLG). A further 4 participants were unable to attend the examinations immediately after interventions (T5), 22 individuals were unable to attend the second follow-up 12 weeks after the end of therapy (T6), and 8 participants missed both follow-up events (T5 + T6) due to illness (11 in total: 3 WBE, 3 LBE, 5 CLG), not being available (2 WBE) or for no reason given (21 in total: 5 WBE, 10 LBE, 6 CLG).

As specified in the study protocol, we selected the last observed carried forward (LOCF) method for missing measurements. As shown in Table 1, sex and age were well balanced between the groups.

### 3.2 Intensity of the training

Participants rated the intensity of the training with the Borg-Scale (6 = no strain, 20 = maximum strain) (Morishita et al., 2021). The mean value (MV) of the mean intensity was 14.0 ±2 in WBE and 14.0 ±2.8 in LBE group. On the first intervention day, they considered the training to be more intense (WBE 14.6 ±2.2; LBE 15.6 ±2.5) than on the last intervention day (WBE 13.4 ±1.7; LBE 13.4 ±2.7). The maximum intensity was rated 15.3 ±1.7 / 15.6 ±1.7 in the WBE and 15.8 ±1.9 / 15.7 ±2.2 in the LBE group on the first and last intervention day, respectively.

### 3.3 Treatment efficacy

#### 3.3.1 Primary outcome parameters

Our primary outcome was the difference in fitness, measured with the 6MRT before (T3) and immediately after interventions (T5). All groups improved between T3 and T5 (*p* < 0.001), with the largest effect in LBE (109 ±113 m, Cohen's d = 0.78), followed by WBE (83 ±82 m, Cohen's d = 0.59) and CLG (58 ±58 m Cohen's d = 0.44). The difference between WBE group and CLG was not significant (*p* = 0.32 in the ITT and *p* = 0.62 in the PP analysis). There was, however, a significant difference between LBE group and CLG (*p* = 0.04 in the ITT and *p* = 0.02 in the PP analysis).

Calculating the effect size (Cohen's d) with the t-test for independent samples for the difference between T5 and T3 in the 6MRT in the ITT population, we found a small, not significant effect in favor of LBE over WBE (Cohen's d = 0.26), a small, not significant effect in favor of WBE over CLG (Cohen's d = 0.35), and a medium effect in favor of LBE over CLG (Cohen's d = 0.56, p = 0.04).

**Fig. 3:**
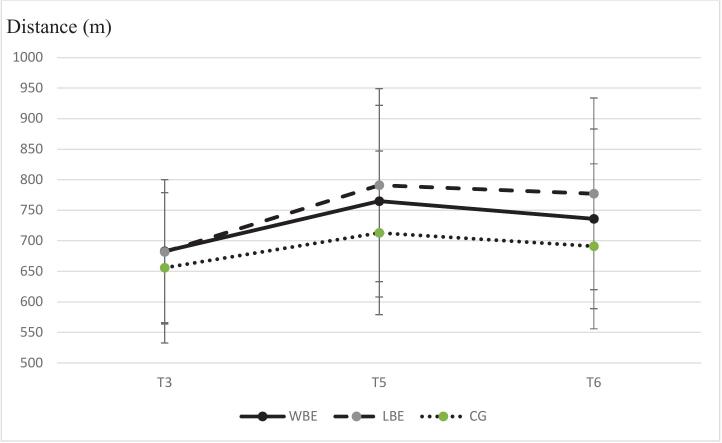
**Six-minute run test at baseline, after intervention and 12 follow-up for water-, land-based exercise, and control group in Germany, 2018.** Bars show standard deviation. *Abbreviations:* T3 baseline, T5 immediately after therapy, T6 12 weeks after therapy, CLG control group, LBE land-based Exercise, WBE water-based exercise.

6MRT at T5 in the WBE group was 765 ±157 m, 791 ±158 m in the LBE group, and 713 ±134 m in the CLG. The difference at T5 between the groups in the ITT-ANCOVA adjusted for baseline was not significant (*p* = 0.12), whereas in the PP analysis the p-value was 0.04. Table 2 and Fig. 3 show the results of the 6MRT at different time points. The LBE group showed the largest improvement in 6MRT, particularly between T3 and T6, while the WBE and CLG groups showed smaller or plateauing gains.

**Table 2:** Six-minute run test analysis of variance and independent *t*-test of water-, land-based exercise, and control group in Germany, 2018.

Time	WBE (m)	LBE (m)	CLG (m)
T3 (mean +/− SD)	683 (±117)	682 (±118)	656 (±123)
T5 (mean +/− SD, p-value^1^)	765 (+/− 157)	791 (±158)	713 (±134)
	0.117		
Difference T5-T3 (mean +/− SD) Cohen's d^3^ (p-value^2^) [95 % CI]	83 (±82)	109 (±113)	58 (±58)
	0.354 (0.316) [51.8; 114.2]	0.559 (0.039) [62.4; 155.6]	
T6 (mean +/− SD, p-value^1^)	736 (±147)	777 (+/− 157) 0.034	691 (+/− 135)
Difference T6-T3 (mean +/− SD) Cohens's d^3^ (p-value^2^) [95 % CI]	53 (±70)	94 (±109)	35 (±51)
	0.28 (0.46) [26.4; 79.6]	0.66 (0.01) [49.0; 139.0]	

1 analysis of variance, ^2^independent t-test (Bray et al., 2016),Cohen's d compared to CLG. *Abbreviations:* m meters, T3 baseline, T5 immediately after therapy, T6 12 weeks after therapy, CLG control group, LBE land-based exercise, SD standard deviation, WBE water-based exercise.

#### 3.3.2 Secondary outcome parameters

At 12 weeks post-therapy (T6), the ITT-ANCOVA (baseline-adjusted) showed *p* = 0.03 (PP *p* = 0.05), driven by a significant LBE vs CLG difference (ITT *p* = 0.01, PP p = 0.02); WBE vs CLG was non-significant (ITT *p* = 0.46, PP *p* = 0.43). BMI, heart rate, and systolic/diastolic BLP differed neither at baseline (T3) nor immediately post-therapy (T5) or at T6 by ANCOVA (see Table 3).We also examined IWQoL and SF-12. In IWQoL, the differences between groups in terms of change between T3 and T5 (*p* = 0.42) and between T3 and T6 (p = 0.12) were statistically not significant. In the body scale of the SF-12 (KROH), the differences between groups in terms of change between T3 and T5 (*p* = 0.40) and between T3 and T6 were also not significant (*p* = 0.34). The difference in the mental scale (PROH) between T3 and T5 was not significant (*p* = 0.456), but between T3 and T6 it showed a *p* = 0.02. Here the difference between WBE and CLG at T6 was 4.41 with a p = 0.01, between LBE and CLG 4.0 with a *p* = 0.57.

**Table 3:** Secondary outcome parameters for water-, land-based exercise, and control group in Germany, 2018.

Parameter	Time point	WBE	LBE	CLG	p^1^
BMI (kg/m^2^)	T3 (SD)	34.8 (±5.7)	33.4 (±4.5)	33.6 (±4.5)	
	T5 (SD)	34.5 (±5.8)	33.1 (±4.5)	33.2 (±4.7)	0.87
	T6 (SD)	34.5 (±5.9)	32.8 (±4.6)	33.1 (±4.9)	0.64
	diff T5-T3 (SD)	−0.3 (±0.71)	−0.4 (±0.87)	−0.5 (±1.28)	
	diff T6-T3 (SD)	−0.3 (±0.86)	−0.6 (±1.33)	−0.6 (±1.9)	
HBR	T3 (SD)	1.0 (±0.07)	1.0 (±0.06)	1.0 (±0.07)	
	T5 (SD)	0.9 (±0.08)	1.0 (±0.06)	1.0 (±0.07)	0.39
	T6 (SD)	0.9 (+/− 0.8)	1.0 (+/− 0.06)	1.0 (±0.07)	0.48
	diff T5-T3 (SD)	0.0 (±0.04)	0.0 (±0.02)	0.0 (±0.04)	
	diff T6-T3 (SD)	0.0 (±0.03)	0.0 (±0.03)	0.0 (±0.04)	
BLP diastolic (mmHg)	T3 (SD)	90 (±14.2)	89 (±7.6)	89 (±10)	
	T5 (SD)	87 (±13)	90 (±10)	88 (±13)	0.69
	T6 (SD)	89 (±11)	89 (±8)	91 (±12)	0.52
	diff T5-T3 (SD)	−2.1 (±13.1)	0.8 (±11)	−0.3 (±12.5)	
	diff T6-T3 (SD)	−0.7 (±13.2)	0.1 (±7.9)	2.6 (±12.5)	
BLP systolic (mmHg)	T3 (SD)	145 (±17.8)	142 (±14.9)	144 (±19.39)	
	T5 (SD)	144 (±14)	146 (±17)	148 (±16)	0.54
	T6 (SD)	141 (±17)	142 (±13)	147 (±20)	0.21
	diff T5-T3 (SD)	−0.9 (±17.6)	3.5 (±16.9)	3.9 (±16.6)	
	diff T6-T3 (SD)	−4.3 (±18.2)	−0.3 (±13.2)	3.1 (±18.1)	
IWQoL	T3 (SD)	72.09 (±16.42)	70.67 (±17.98)	67.41 (+/− 23.56)	
	T5 (SD)	74.42 (+/− 16.09)	75.52 (+/− 18.61)	71.71 (+/− 23.99)	0.42
	T6 (SD)	73.80 (+/− 17.19)	76.72 (+/− 18.80)	73.20 (+/− 22.88)	0.12
	diff T5-T3 (SD)	2.33 (±7.58)	4.84 (±7.09)	3.89 (±7.06)	
	diff T6-T3 (SD)	1.71 (±8.88)	6.05 (±8.37)	5.79 (±7.20)	
SF-12 KROH	T3 (SD)	−14.36 (+/− 9.08)	−11.91 (+/− 8.66)	−12.50 (+/− 8.80)	
	T5 (SD)	−13.08 (+/− 8.06)	−13.19 (+/− 9.01)	−10.83 (+/− 8.49)	0.40
	T6 (SD)	−14.59 (+/− 7.94)	−14.05 (+/− 9.77)	−11.34 (+/− 7.91)	0.34
	diff T5-T3 (SD)	1.28 (±9.43)	−1.28 (±8.62)	1.67 (±6.06)	
	diff T6-T3 (SD)	−0.23 (±9.87)	−2.13 (±9.49)	1.15 (±6.70)	
SF-12 PROH	T3 (SD)	−17.66 (+/− 7.73)	−19.00 (+/− 6.81)	−17.51 (+/− 7.68)	
	T5 (SD)	−16.73 (+/− 7.70)	−19.47 (+/− 7.14)	−18.09 (+/− 7.94)	0.46
	T6 (SD)	−15.68 (+/− 8.68)	−19.68 (+/− 6.41)	−20.09 (+/− 5.84)	0.02
	diff T5-T3 (SD)	0.93 (±6.10)	−0.47 (±5.86)	−0.58 (±5.69)	
	diff T6-T3 (SD)	1.98 (±8.66)	−0.86 (±5.22)	−2.58 (±7.23)	
HbA1c	T3 (SD)	38.3 (±4.3)	39.7 (±6.5)	40.8 (±7.7)	
	T5 (SD)	37.8 (±4.8)	39.4 (±5.9)	40 (±8.2)	0.33
	diff T5-T3 (SD)	−0.4 (±1.2)	−0.4 (±1.2)	−0.78 (±1.8)	
TRG	T3 (SD)	200.5 (±96.4)	194.6 (±90.4)	217.1 (±230.7)	
	T5 (SD)	190 (±113)	211 (±109)	299 (±123)	0.43
	diff T5-T3 (SD)	−10.2 (±56.1)	16.7 (±104.7)	−17.6 (±137)	
Cholesterol	T3 (SD)	209.1 (±28.7)	210.6 (±33.7)	208.2 (±35.6)	
	T5 (SD)	205 (±29.6)	214.1 (±38.5)	210.1 (±38.5)	0.23
	diff T5-T3 (SD)	−3.9 (±16.9)	3.5 (+/− 20.6)	1.9 (+/− 23.4)	
HDL	T3 (SD)	55.1 (±18.5)	54.4 (±14.3)	53.9 (±14.2)	
	T5 (SD)	57 (±19.6)	55 (±13.1)	56.1 (±14.2)	0.11
	diff T5-T3 (SD)	1.8 (±6.1)	0.6 (±6.7)	2.1 (±5.8)	
LDL	T3 (SD)	137.6 (±22.6)	139.7 (±29.3)	135.9 (±35.7)	
	T5 (SD)	137.2 (±25)	147.6 (±38.2)	141.3 (±35.5)	0.31
	diff T5-T3 (SD)	−0.38 (±16.1)	7.9 (±22.1)	5.2 (±15)	

1 P-value based on analysis of variance with baseline values (T3) as covariates. *Abbreviations:* T3 baseline, T5 immediately after therapy, T6 12 weeks after therapy, BMI body mass index, BLP blood pressure, CLG control group, diff difference, HBR hip-belly ratio, IWQoL Impact of Weight on Quality of Life-lite, KROH body scale of the SF-12, LBE land-based Exercise, PROH mental scale of the SF-12, SF-12 12-Item Short-Form Health-Survey, TRG triglycerides, HDL high density lipoproteins, LDL low densitiy lipoproteins, WBE water-based exercise.

With regard to the preference for one of the interventions, it was found that 82 of the 87 individuals expressed a preference for the WBE group, with an average score of 4.4 out of 5. Four participants indicated a preference for the LBE group, while one individual selected the CLG. With respect to motivation, the mean scores for fitness, weight reduction, and participation in a study were 4.72, 4.63, and 3.31, respectively.

### 3.4 Safety

From T3 to T6, 104 adverse events (AE) occurred: 2 pre-training (T3–T4), 69 during training (T4–T5) and 33 post-training (T5–T6). During training, AE counts were similar across groups—WBE: 24, LBE: 20, CLG: 25. Flu-like symptoms were most common (WBE 16, LBE 11, CLG 10), and three bladder infections occurred only in the CLG. Orthopedic issues (knee, hip, shoulder, back pain) affected three participants in each exercise group and two in the CLG. The remaining 24 training-period AE were mainly household/recreational accidents (e.g., cuts, burns, one ACL rupture) and surgeries (e.g., maxillofacial, urologic, gynecologic; plus one elective cardiac cath in CLG).

## 4 Discussion

The study compared WBE and LBE effects on fitness (6MRT), cardiovascular risk factors, compliance, and adverse events. After 10 weeks, WBE and LBE showed no differences in fitness, blood pressure, BMI, HRV, QoL, or SF-12, but only LBE outperformed control on the 6MRT at T5 and T6 (*p* < 0.05). All groups improved fitness, and per-protocol results mirrored ITT findings. Adverse events were similar, though colds were slightly more common in WBE.

WBE showed smaller improvements than LBE on the 6MRT, without reaching statistical significance and did not demonstrate superiority to CLG. A lack of motivation was unlikely to have been a factor, given that the majority of participants expressed a preference for WBE. I. Although prior studies suggest WBE advantages, our data did not confirm those differences (Lopera et al., 2016; Nosrani et al., 2023; Zhu, 2023; Miguet et al., 2023; Liao et al., 2024; Saquetto et al., 2019; Ruangthai et al., 2020; Sukkee, 2016; Soori et al., 2017). Recent studies highlight the cardiovascular and metabolic benefits of WBE, including improvements in endothelial function, inflammatory markers, and mental well-being (Nosrani et al., 2023; Zhu, 2023). For example, WBE has been associated with a greater reduction in body fat percentage, while LBE tends to improve abdominal muscle strength and social or psychosocial parameters. Additionally, aquatic exercises have demonstrated the potential to mitigate energy compensatory responses without negatively affecting energy balance (Miguet et al., 2023).

Short-term high-intensity aquatic interval training has shown similar or superior effects on physiological markers such as lean body mass and vital capacity compared to land-based protocols, supporting the efficacy of WBE in targeted populations (Liao et al., 2024). Other studies have reported slight advantages of WBE over LBE in terms of blood lipid levels, with comparable effects on blood pressure and fitness, depending on the intervention duration and frequency (Nosrani et al., 2023; Ruangthai et al., 2020). Furthermore, research suggests that WBE may reduce systemic inflammation and improve microvascular health, which are particularly relevant for aging and at-risk populations (Liao et al., 2024). Both WBE and LBE offer significant health benefits, though their effects differ depending on the focus of the intervention. In multidisciplinary programs combining psychological, educational, and nutritional components, WBE was more effective at reducing body fat, while LBE excelled in enhancing muscle strength and psychosocial outcomes, suggesting complementary benefits (Lopera et al., 2016). Similarly, a longer 24-week intervention showed slight advantages of WBE in improving lipid profiles, whereas both modalities were equally effective in lowering blood pressure and enhancing fitness, highlighting the broader cardiovascular benefits of both approaches (Ruangthai et al., 2020).

Structured and guided WBE programs also appeared more effective compared to unstructured LBE activities, emphasizing the importance of intervention quality (Sukkee, 2016). Aerobic and combined WBE further demonstrated superior outcomes in targeted physiological measures when compared to LBE delivered via dance or gymnastics, underscoring the potential of WBE for specific populations (Silva, 2018). A small study found WBE and combined WBE-muscle training improved BMI and fat percentage more than muscle training alone, reinforcing the need for larger studies to confirm these results (Soori et al., 2017). A recent study on women with breast cancer found no significant differences between LBT and WBT. However, WBT might be more effective in increasing physical activity levels than LBT in this population (Mur-Gimeno, n.d.).

A possible, but hypothetical reason for the unexpected outcome may be that the 6MRT was inappropriate for measuring physical fitness after WBE. We acknowledge that the 6MRT may inherently favor land-based adaptations, since WBE engages different muscle groups and buoyancy reduces weight-bearing. As WBE activates other muscle groups than LBE, LBE is more likely to train the muscles used for walking. The 6MRT might have favored LBE. While our sample size was powered to detect a 15 % (≈90 m) difference in 6MRT, the observed between-group difference of 25 m falls below the 30–50 m MCID threshold; accordingly, we interpret the WBE versus control improvements as statistically non-significant and of limited clinical relevance.

Strengths of our study were the randomized, controlled, pragmatic (training sessions performed by local qualified providers) design, the specified primary outcome (6MRT), and comprehensive testing of relevant secondary outcomes. The intensity of the training was documented, but according to the pragmatic design not predefined. Further strengths were the successful randomization (no difference in baseline characteristics between the groups) and a similar intensity of training in the two intervention groups.

The primary limitation of our study is the high dropout rate. Specifically, 12 participants withdrew during the trial, 12 missed the follow-up at time point T5, and 30 missed T6 (with 8 participants missing both). This significantly affects the validity of the results, particularly at T6. Despite using LOCF to preserve sample size, caution is warranted when interpreting results at this timepoint due to the risk of bias from non-random attrition. However, although all therapists followed a standardized manual and received identical training and supervision, we did not formally assess therapist fidelity or include therapist identity as a covariate in the analysis. Manuscript preparation and additional predefined analyses were delayed by COVID-19–related disruptions to institutional access and research workflows; recruitment and data collection occurred in July–September 2018. Future research in the group of physically inactive overweight individuals should include other water-specific training forms tests (e.g., underwater shuttle) to capture modality-specific adaptations. Stratifying participants by baseline fitness and extending follow-up will help determine if cardiopulmonary gains are maintained equally in WBE and LBE. The risk of a high dropout rate should be targeted by intensified care and”motivation“.

## 5 Conclusion

Both WBE and LBE improved fitness and cardiometabolic markers, but only LBE outperformed control on the 6-min run test. Secondary outcomes—BMI and quality of life—were similar. Since effects on cardiovascular risk factors didn't differ and many participants preferred WBE, program choice should consider individual motivation, physical limitations (e.g., joint pain), and facility access. Tailoring exercise to participant needs may boost adherence and effectiveness. Further research should examine modality-specific fitness outcomes.

## CRediT authorship contribution statement

**Vanessa Maria Eichel:** Writing – review & editing, Validation, Resources, Methodology, Investigation. **Katja Oomen-Welke:** Writing – original draft, Investigation, Formal analysis. **Maria Störk:** Resources, Methodology, Investigation, Formal analysis, Data curation. **Roman Huber:** Writing – review & editing, Supervision. **Maximilian Andreas Storz:** Writing – review & editing, Methodology, Investigation, Formal analysis, Data curation. **Johannes Naumann:** Validation, Supervision, Methodology, Investigation, Formal analysis, Data curation, Conceptualization.

## Informed consent statement

Informed consent was obtained from all individuals involved in the study.

## **Institutional review board statement**

The study was conducted in accordance with Good Clinical Practice Guidelines (CPMP/ICH/135/95; Topic E6 (R1); and GCP-V), the Declaration of Helsinki and local laws. The protocol was reviewed and approved by the Ethics Committee of Albert-Ludwigs-Universität Freiburg (EK-Freiburg 25/18). The study was prospectively registered in the European Clinical Trials Database (**DRKS00014004**), date of registration 31/07/2018.

## Declaration of competing interest

The authors declare that they have no known competing financial interests or personal relationships that could have appeared to influence the work reported in this paper.

## Data Availability

The authors confirm that the data supporting the findings of this article are available within the article.
